# Genome editing methods in animal models

**DOI:** 10.1080/19768354.2020.1726462

**Published:** 2020-02-17

**Authors:** Hyunji Lee, Da Eun Yoon, Kyoungmi Kim

**Affiliations:** a Center for Genome Engineering, Institute for Basic Science, Daejeon, Republic of Korea; b Department of Biomedical Sciences, Korea University College of Medicine, Seoul, Republic of Korea; c Department of Physiology, Korea University College of Medicine, Seoul, Republic of Korea

**Keywords:** CRISPR-Cas9 system, genome editing, animal model, *In vivo* delivery

## Abstract

Genetically engineered animal models that reproduce human diseases are very important for the pathological study of various conditions. The development of the clustered regularly interspaced short palindromic repeats (CRISPR) system has enabled a faster and cheaper production of animal models compared with traditional gene-targeting methods using embryonic stem cells. Genome editing tools based on the CRISPR-Cas9 system are a breakthrough technology that allows the precise introduction of mutations at the target DNA sequences. In particular, this accelerated the creation of animal models, and greatly contributed to the research that utilized them. In this review, we introduce various strategies based on the CRISPR-Cas9 system for building animal models of human diseases and describe various *in vivo* delivery methods of CRISPR-Cas9 that are applied to disease models for therapeutic purposes. In addition, we summarize the currently available animal models of human diseases that were generated using the CRISPR-Cas9 system and discuss future directions.

## Introduction

The clustered regularly interspaced short palindromic repeats (CRISPR) system, which is derived from the adaptive immune system of prokaryotes, is a breakthrough genome editing technology that can be applied to a variety of researches. Among them, the third-generation gene editing system *Streptococcus pyogenes* Cas9 (SpCas9) can efficiently introduce mutations at desired target positions in the genome in a guide RNA (gRNA)-dependent manner (Cong et al. 2013). The target recognition sequence of SpCas9, called the protospacer-adjacent motif (PAM) sequence, is 5′–NGG–3′ (N = A or T or G or C). To form a functional Cas9/gRNA complex, SpCas9 requires a gRNA consisting essentially of a CRISPR RNA (crRNA) and a trans-activating crRNA (tracrRNA). The Cas9 protein, which can target specific genes for editing and correction, generates DNA double-strand breaks at 20 base pair of target sequence positions that are complementary to the short gRNAs. The cleaved DNA is repaired by non-homologous end joining (NHEJ) or homology directed repair (HDR) endogenous repair mechanisms, to produce insertion or deletion (indel) mutations.

SpCas9 is widely used in a variety of basic research and clinical applications, including *in vivo* studies, because it provides highly efficient and characterized PAM sequences. Other highly efficient *in vivo* gene editing tools include *Acidaminococcus sp.* Cpf1 (AsCpf1), *Lachnospiraceae bacterium* Cpf1 (LbCpf1), *Staphylococcus aureus* Cas9 (SaCas9), and *Campylobacter jejuni* Cas9 (CjCas9). Among them, AsCpf1 and LbCpf1 have high HDR efficiencies, which allow the insertion or conversion of specific sequences. Therefore, these tools can be useful to generate knockin animal models (Moreno-Mateos et al. 2017). Moreover, SaCas9 and CjCas9 are smaller than SpCas9 and can be effectively used in *in vivo* delivery studies using adeno-associated virus (AAV) with packing limitation (Ran et al. 2015; Kim et al. 2017). Recently, cytosine base editor (CBE) and adenine base editor (ABE) have been reported, which are new programable base editing methods that can convert C to T or A to G at the nucleotide level (Komor et al. 2016; Gaudelli et al. 2017). Base editors are useful gene editing methods that can directly apply human pathogenic single-nucleotide polymorphisms (SNPs) to animal models and therapeutic studies. Here, we describe various advanced genome editing methods that can be applied to animal modeling and therapeutic research, and discuss future prospects.

## Generation of animal models of human diseases using genome editing methods (Table 1)

**Figure 1.**
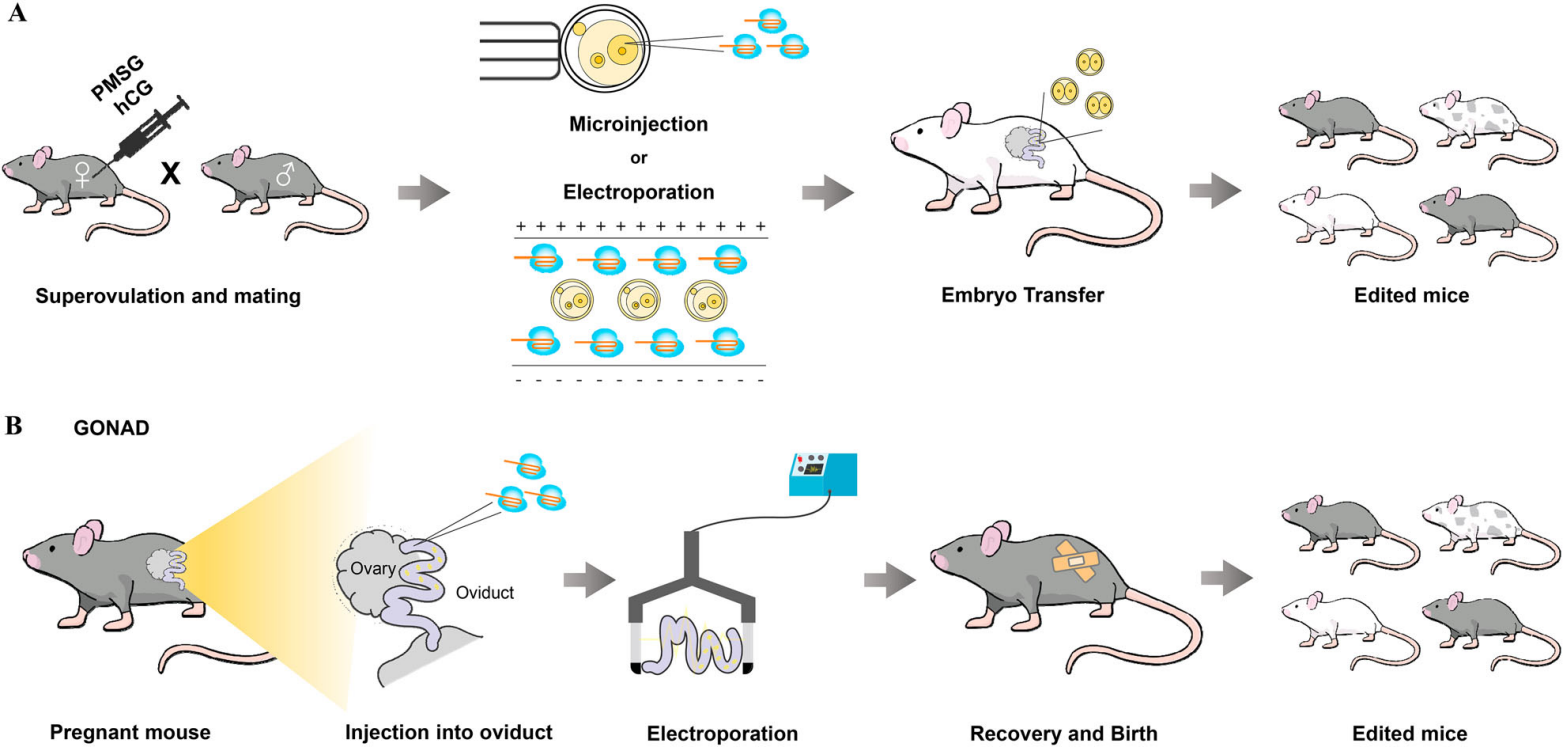
**Mouse modeling methods using the CRISPR system.** A, CRISPR delivery to zygote embryos using microinjection (pronucleus/cytoplasm) or electroporation. Edited 2-cell-stage embryos are transplanted into a surrogate mouse and the edited offspring are obtained. B, Genome editing via oviductal nucleic acid delivery (GONAD) is a new method of introduction of the Cas9/gRNA complex into embryos. Direct injection of the Cas9/gRNA complex for genome editing into oviduct of pregnant mouse, followed by an electrical impulse.

The use of fertilized 1-cell-stage embryos is the most common method of producing genome-engineered animal models. The methods that are used for producing animal models using fertilized embryos with the CRISPR system include microinjection, electroporation, and genome editing via oviductal nucleic acid delivery (GONAD) (Figure 1). Microinjection is a method of injecting the Cas9/gRNA complex directly into the cytoplasm or pronucleus of fertilized 1-cell embryos, whereas electroporation enables gene editing by inducing electric stimulation in the presence of the Cas9/gRNA complex to fertilized 1-cell embryos (Wang et al. 2013; Qin et al. 2015). GONAD is a new method to introduce the Cas9/gRNA complex into embryos without sacrificing animals (Gurumurthy et al. 2016). The Cas9/gRNA complex for genome editing is injected directly into the oviduct of pregnant mouse, followed by *in situ* electroporation. Here, we describe studies of animal models of diseases using various CRISPR-based genome editing methods.

**Table 1.** Generation and treatment of animal models of human diseases using genome editing methods.

Species	Target gene	Disease	Technique	Editing Method		Reference
Generatiof disease animal models						
Mouse	Fah	Tyrosinemia	Microinjection	SpCas9	mRNA	Li et al. (2015)
	Rag1, IL2RgammaC	Immunodeficient				
	Notch3	Lateral meningocele syndrome	Microinjection	SpCas9	mRNA	Canalis et al. (2018)
	ATP6V1H	Osteoporosis	Microinjection	SpCas9	mRNA	Duan et al. (2016)
	Bril	Osteogenesis imperfecta (OI)	Microinjection	SpCas9	mRNA	Rauch et al. (2018)
	Sox9	Acampomelic campomelic dysplasia (ACD), Campomelic dysplasia (CD)	Microinjection	SpCas9	mRNA	Mochizuki et al. (2018)
	Dystropin	Duchenne Muscular Dystrophy (DMD)	Electroporation	Cytidine Base editor 3 (BE3)	RNP	Kim et al. (2017)
	p53, Lkb1, KRAS	Cancer	Intratracheal injection	SpCas9	AAV	Platt et al. (2014)
Rat	Tyrosine hydroxylase (TH)	Parikinson’s disease (PD)	Intracranial injection	SpCas9	AAV	Back et al. (2019)
pig	Huntingtin (HTT)	Huntington’s disease (HD)	Somatic cell nuclear transfer	SpCas9	plasmid	Yan et al. (2018)
	Parkin, Pink1	Parikinson’s disease (PD)	Somatic cell nuclear transfer	SpCas9	plasmid	Zhou et al. (2015)
Monkey	Dystropin	Duchenne Muscular Dystrophy (DMD)	Microinjection	SpCas9	mRNA	Chen et al. (2015)
Dog	Myostatin	Muscle hypertrophy	Microinjection	SpCas9	mRNA	Zou et al. (2015)
Rabbit	Myostatin	Muscle hypertrophy	Microinjection	SpCas9	mRNA	Lv et al. (2016)
	Dystropin	Duchenne Muscular Dystrophy (DMD)	Microinjection	SpCas9	mRNA	Sui et al. (2018)
	PAX4	Diabetes mellitus (DM)	Microinjection	SpCas9	mRNA	Xu et al. (2018)
Treatment of disease animal models						
Mouse	F8	Hemophilia A	Patient-Derived iPSCs correction and transplantion	SpCas9	Plasmid	Park et al. (2015)
	F9	Hemophilia B	Intravenous injection	SaCas9	AAV	Ohmori et al. (2017)
	Dystropin	Duchenne Muscular Dystrophy (DMD)	Intramuscular injection,	SpCas9	AAV	Tabebordbar et al. (2016)
			Intraperitoneal injection,	SaCas9	AAV	Nelson et al. (2016)
			Intravenous injection,	SpCas9	AAV	Long et al. (2016)
			Retro-orbital injection	SpCas9	AAV	Bengtsson et al. (2017)
			Intramuscular injection	SpCas9	RNP with gold nanoparticle	Li et al. (2015)
			Intramuscular injection	Adenine Base Editor (ABE)	AAV	Ryu et al. (2018)
	SOD1	Amyotrophic lateral sclerosis (ALS)	Intravenous injection	SaCas9	AAV	Gaj et al. (2017)
	Fah	Tyrosinemia	Intravenous injection(Hydrodynamic injection)	SpCas9	Plasmid	Yin et al. (2014)
			Intravenous injection	SpCas9	AAV	Yin et al. (2016)
			Intravenous injection (Hydrodynamic injection)	Adenine Base Editor (ABE)	Plasmid	Song et al. (2019)
	LTR, Gag, Pol	HIV-1/AIDS	Intravenous injection,	SaCas9	AAV	Kaminski et al. (2016)
			Intravaginal injection,			Yin et al. (2017)
			Retro-orbital injection			
	Huntingtin (HTT)	Huntington’s disease (HD)	Stereitactic injection	SpCas9	AAV	Monteys et al. (2017)
	Vegfr2	Age-related macular degeneration (AMD)	Intravitreal injection	SpCas9	AAV	Huang et al. (2017)
	Vegfr		Subretinal injection	SpCas9	RNP with liposome	Kim et al. (2017)
	Vegfa		Intravitreal injection	CjCas9	AAV	Kim et al. (2017)
	Hif1a		Intravitreal injection	LbCpf1	AAV	Koo et al. (2018)
	Bace1	Alzheimer’s disease (AD)	Intraacranial injection	SpCas9	RNP with peptide	Park et al. (2019)
Rat	Rho	Retinal dystropy	Subretinal injection	SpCas9	plasmid	Bakondi et al. (2016)
Dog	Dystropin	Duchenne Muscular Dystrophy (DMD)	Intramuscular injection, Intravenous injection	SpCas9	AAV	Amoasii et al. (2018)
Pig	Alb	Liver failure, traumatic shock	Microinjection	SpCas9	mRNA	Peng et al. (2015)

### Mice and rats

The most common animal model of diseases generated using genome editing is the rodent, including mice and rats. In 2013, the Jaenisch group reported knockin or knockout mouse models that were generated using a one-step method via cytoplasmic microinjection of fertilized eggs using the RNA-guided Cas9 nuclease system (Wang et al. 2013; Mashiko et al. 2013; Shen et al. 2013). Since then, various animal models of disease have been generated using the CRISPR-Cas9 system.

The CRISPR-Cas9 system allows the generation of mutant mouse models that cannot be genetically manipulated using previous approaches. The Su group reported the generation of an immunodeficient mouse model with *NRG* (NOD-Rag1-/-IL2RgammaC-null) knockout and a tyrosinemia mouse model with *Fah* gene knockin by combining *in vitro* fertilization (IVF) with the CRISPR-Cas9 technology. A sufficient number of fertilized embryos were produced through IVF, and a high rate of gene targeting was achieved via pronuclear microinjection of the Cas9 mRNA, gRNA, and single-stranded oligonucleotide DNA (ssDNA) into the embryos (Li et al. 2015).

One group created a human lateral meningocele syndrome (LMS)-related mutant mouse model of the *Notch3* gene via microinjection of the Cas9 mRNA and gRNA. The LMS-related mutant mouse model induced a 35%–60% decrease in the cancellous bone volume, together with a reduction in trabecular number, which mimics the skeletal manifestation of lateral meningocele syndrome (Canalis et al. 2018). In addition, several studies have reported mouse models of osteoporosis that were generated by knocking out the *ATP6V1H* gene, a subunit of V-ATPase that plays an important role in the biological and physiological functions of osteoclasts (Yao et al. 2007; Duan et al. 2016; Zhang et al. 2017). The Moffatt group reported a knockin mouse model that mimicked the *BRIL* mutation of patients with osteogenesis imperfecta type V perfectly. Osteogenesis imperfecta type V is caused by an autosomal dominant c.-14C > T mutation in the 5′ untranslated region (UTR) of *BRIL*, which creates a novel translational start site that adds five residues (MALEP) in frame to the natural sequence of BRIL. The authors synthesized gRNAs targeting the 5′UTR of the mouse *Bril* gene and injected them into mouse embryos together with the Cas9 mRNA and a 67mer single-stranded oligodeoxynucleotide (ssODN) carrying the c.-14C > T mutation. MALEP-BRIL heterozygous mice also induced less mineralization and excessive cartilaginification from incomplete osteoblast differentiation and bone collar formation (Rauch et al. 2018).

In humans, SRY-box 9 (*SOX9*) mutations lead to acampomelic campomelic dysplasia (ACD) and campomelic dysplasia (CD), which are developmental disorders of cartilage. The Takai group generated a mouse model of ACD and CD by deleting the cartilage-specific *SOX9* enhancer, termed the rib-cage-specific enhancer, using the embryo microinjection method of the CRISPR system. This model was consistent with the clinical phenotypes of human ACD/CD patients and is expected to be applicable to studies aimed at the treatment of ACD/CD (Mochizuki et al. 2018).

As mentioned previously, the Liu group introduced a new CBE method that can convert C to T at the nucleotide level (Komor et al. 2016). The Kim group reported a mouse model of Duchenne muscular dystrophy (DMD) that was generated by inducing stop codons from a single C to T conversion at exon 20 of the *dystrophin* gene via cytidine-deaminase-mediated base editing by embryo electroporation using a CBE protein and gRNA (Kim et al. 2017).

The Zhang groups reported CRISPR-Cas9 knockin mice for genome editing and cancer modeling. Using this model, a gRNA was delivered to the AAV vector system to model simultaneously the dynamics of the top three significantly mutated genes, i.e. *KRAS*, *p53*, and *LKB1*, to cause lung adenocarcinoma (Platt et al. 2014).

As for rats, the Harvey group generated a Parkinson’s disease model by knocking out Tyrosine Hydroxylase (*TH*) gene from brain dopaminergic neurons in rats via intracranial injection of AAV vectors expressing SpCas9 and gRNA (Back et al. 2019). This Parkinson’s disease rat model is expected to be used for multipurpose studies aimed at developing therapies for this disease.

### Large animals (pigs, monkeys, dogs, and rabbits)

Various neurodegenerative diseases, including Alzheimer’s disease (AD), Huntington’s disease (HD), Parkinson’s disease (PD), and amyotrophic lateral sclerosis (ALS), share the characteristics of age-dependent neurological symptoms and selective neuronal degeneration (Wyss-Coray 2016). HD results from a monogenetic mutation consisting of the expansion of a CAG repeat located in exon 1 of the huntingtin (*HTT*) gene (Bates et al. 2015). One group used the CRISPR-Cas9 system to generate a HD pig model that expresses full-length mutant HTT endogenously. In that study, the brains of the HD model pigs showed selective degeneration of striatal medium spiny neurons (Yan et al. 2018).

The loss of function of the Parkin and PINK1 proteins can cause PD. PD pig models have been generated by editing the *Parkin*, *Pink1*, and *DJ1* genes using CRISPR-system-based somatic cell nuclear transfer (Hai et al. 2014; Zhou et al. 2015).

Another study reported a monkey model of DMD by targeting CRISPR-Cas9 to exons 4 and exons 46 of the *dystrophin* gene (Chen et al. 2015; Niu et al. 2014).

The *myostatin* (*MSTN*) gene is a negative regulator of skeletal muscle mass (McPherron et al. 1997). MSTN knockout dog and rabbit models with a double muscle phenotype were generated by injecting the Cas9 mRNA and gRNA into zygotes, to generate a muscle hypertrophy model (Zou et al. 2015; Lv et al. 2016). One group also reported the generation of DMD rabbit models via microinjection of Cas9 mRNA and gRNA targeting dystrophin exon 51 into rabbit zygotes (Sui et al. 2018).

Lastly, *PAX4* is a major diabetes mellitus (DM) susceptibility gene that is related with many different types of DM, including T1DM, T2DM, maturity-onset diabetes, type 9 (MODY9), and ketosis-prone diabetes. A *PAX4* knockout model was generated by co-injection of the Cas9 mRNA and gRNA into rabbit zygotes. This rabbit model of DM exhibited typical phenotypes of growth retardation, persistent hyperglycemia, reduced insulin-producing b cells, and increased glucagon-producing cells (Xu et al. 2018).

## Therapies for animal models of human diseases using genome editing methods (Table 1)

**Figure 2.**
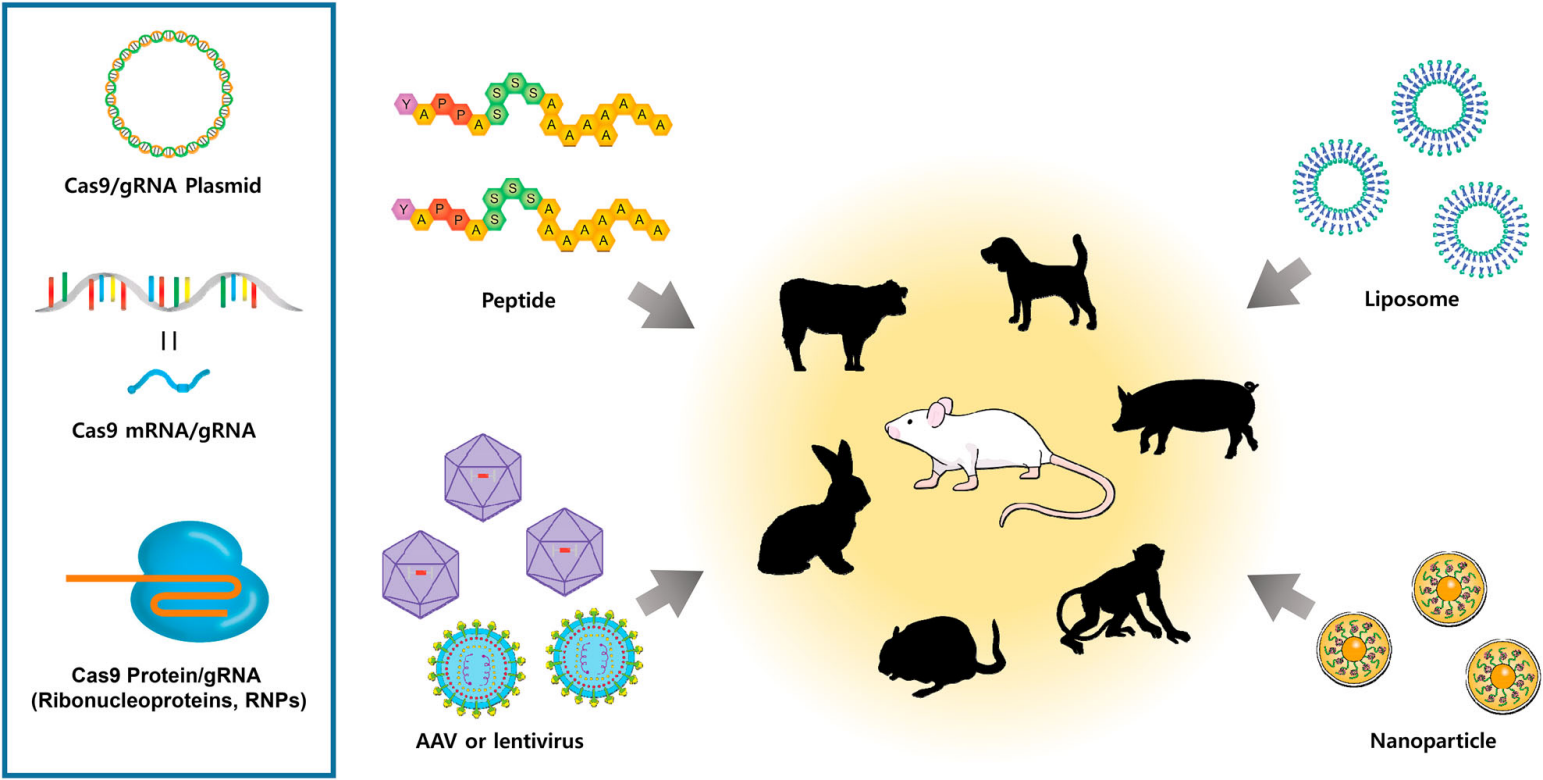
**Strategies for delivering the CRISPR system *in vivo*.** The CRISPR-Cas9 system can be delivered in either DNA, mRNA, or protein form *in vivo*, to induce gene editing. *In vivo* delivery methods, such as viruses, liposomes, peptides, or nanoparticles, have been developed for genome editing and can be applied to a variety of animals.

The CRISPR delivery system for genome editing is classified into plasmid-based CRISPR-Cas9, Cas9 mRNA/gRNA, and Cas9/gRNA ribonucleoproteins (RNPs). The delivery strategies of the CRISPR-Cas9 gene editing system for therapeutic applications include the use of peptides, liposomes, or nanoparticles as carriers or the use of viral delivery systems (such as AAV and lentiviruses) (Figure 2). Here, we introduce a series of studies aimed at developing therapies for animal models of human diseases using genome editing methods.

### Mice and rats

The Kim group reported functional correction of *F8* gene chromosomal inversions in iPSCs derived from patients with hemophilia A using CRISPR-Cas9 (Park et al. 2015). Similarly, hemophilia B was also corrected by *F9*-gene-targeted SaCas9 AAV in mice (Ohmori et al. 2017).

DMD is an X-linked recessive disease that is caused by mutations in the *dystrophin* gene, which contains 79 exons, resulting in progressive muscle degeneration, muscle weakness, and myopathy (Hoffman et al. 1987). Most attempts at DMD correction aimed to restore the reading frame of the *DMD* locus via deletion of the exon(s) in which a pathogenic variant was located. Recently, many groups used the DMD mouse model to demonstrate the therapeutic potential of CRISPR-Cas9-mediated genome editing. Several groups restored dystrophin expression in a DMD mouse model (mdx) harboring a frameshift mutation in exon 23 of the gene by genome editing using an AAV delivery system of SaCas9 or SpCas9 with gRNA (Long et al. 2016; Nelson et al. 2016; Tabebordbar et al. 2016; Bengtsson et al. 2017). In addition to AAV, treatments that deliver RNP–gold particle complexes have also been reported (Lee et al. 2017). *Dmd* knockout mice were used by the Kim group to inject trans-splicing viral vectors (tsAAVs) of the ABE into the tibialis anterior muscle, to convert a premature stop codon to a glutamine codon and restore the expression of the dystrophin protein (Ryu et al. 2018).

ALS, characterized by loss of motor function, is a fatal and incurable neurodegenerative disease affecting the spinal cord and brain. Approximately 20% of familial cases of ALS carry autosomal dominant mutations in the superoxide dismutase 1 (*SOD1*) gene. The Schaffer group demonstrated that disruption of mutant SOD1 expression in the G93A-SOD1 mouse model of ALS via *in vivo* CRISPR/Cas9 genome editing using an AAV vector resulted in delayed disease onset, improved motor function, and reduced muscle atrophy (Gaj et al. 2017).

Hereditary tyrosinemia type I (HTI) is a fatal genetic disease caused by a mutation in fumarylacetoacetate hydrolase (FAH), the last enzyme of the catabolic pathway. FAH deficiency is associated with the accumulation of fumarylacetoacetate; accumulation of toxic metabolites in hepatocytes leads to severe liver damage and renal proximal tubule damage. The Fah5981SB mouse model, which harbors a homologous G to A point mutation in the last nucleotide of exon 8 of the *Fah* gene, recapitulates the human disease fully (Paulk et al. 2010). Recent studies have shown that the application of plasmid delivery of SpCas9 or ABE, gRNA, and HDR template DNA using the hydrodynamic injection method induced specific genomic correction of the *Fah* splicing mutation (Yin et al. 2014; Song et al. 2019; Yin et al. 2016). Another group reported that the application of a combination of viral and lipid nanoparticle-mediated systems of delivery of the SpCas9 mRNA, gRNA, and repair template DNA enables specific correction of the *Fah* splicing mutation in a Fah5981SB mouse model (Yin et al. 2016).

Complete elimination or sterilization of human immunodeficiency virus (HIV) is very important to achieve permanent treatment for HIV/acquired immunodeficiency syndrome. The Khalili and Hu groups removed the HIV-1 DNA via intravenous injection of an AAV system containing gRNA and SaCas9 into a humanized mouse model of chronic HIV-1 infection (Yin et al. 2017; Kaminski et al. 2016). This approach offers the potential to be used as a tool to remove targeted fragments of the HIV-1 genome from latently infected human cells.

Huntington disease is a genetic degenerative neurological disease caused by the expansion of CAG repeats in the *huntingtin* gene. The Davidson group reported a study of the therapeutic application of gene editing of mutant *HTT* alleles using the Cas9/gRNA AAV system in a transgenic HD mouse model harboring the human allele (Monteys et al. 2017).

Targeting of various angiogenesis-related genes has been reported as a therapy for age-related macular degeneration. For example, Vegfr2 targeting by AAV intravitreal injection (Huang et al. 2017), Vegfr targeting by RNP and liposome subretinal injection (Kim et al. 2017), and Vegfa targeting using CjCas9 AAV intravitreal injection (Kim et al. 2017) have been reported. In addition, a recent study reported the targeting of the *HIF1* gene using LbCpf1 AAV (Kim et al. 2017) in a laser-injury-induced mouse model of choroidal neovascularization (CNV).

Regarding treatments for AD, one group recently reported using Cas9/gRNA RNPs and R7L10 peptide nano-complexes to target the *Bace1* gene to alleviate the symptoms of AD in 5xFAD and APP knockin mice (Park et al. 2019).

We also address the development of therapeutic approaches using CRISPR-Cas9 in rat models of human diseases. Transgenic S334ter rats carrying the mouse genomic fragment containing RhoS334 exhibit phenotypic similarities to human class-I RHO mistrafficking mutations and show continual photoreceptor (PR) loss and commensurate vision decline. The resultant peptide (RHOS334) lacks three serine residues that are required for PR deactivation after light stimulation and part of the signal sequence that is required for RHO trafficking to photoreceptor outer segments (Sung et al. 1994). The Wang group showed that CRISPR-Cas9 can be used *in vivo* to ablate selectively the *rhodopsin* gene carrying the dominant S334ter mutation (RhoS334) in a rat model of severe autosomal dominant retinitis pigmentosa. Allele-specific destruction of RhoS334 was induced by electroporation after subretinal injection of a Cas9/gRNA plasmid, which prevented retinal degeneration and improved visual function (Bakondi et al. 2016).

### Large animals (dogs and pigs)

The delta E50-MD canine model of DMD, with loss of exon 50 and skipping of exon 51, carries a mutation corresponding to the mutant ‘hotspot’ in the human *dystrophin* gene. The Olson group delivered CRISPR gene editing components to dogs via intramuscular or systemic delivery using the AAV system. After systemic delivery to skeletal muscle, the levels of dystrophin were recovered to up to 90% of the normal values. These data gleaned from large-animal models support the concept that gene editing approaches can be clinically useful in the treatment of DMD (Amoasii et al. 2018).

In the pig model, the therapy approach is slightly different from that used for other animal models. Human serum albumin (HAS) has therapeutically significance for patients with liver failure and traumatic shock; however, its high cost and low availability make clinical use difficult. As a result, transgenic pigs were generated as a source of human serum albumin, but the purification of the human albumin only from endogenous pig albumin presented a practical challenge. The Zhang group used CRISPR-Cas9-mediated gene editing to replace the pig albumin gene with the human albumin cDNA (Peng et al. 2015). This group created pigs that only produced recombinant human albumin, which provides a promising strategy for the production of other biomedical therapeutics in large domesticated animals.

## Conclusion

The CRISPR-Cas9 system is a very important and useful gene editing tool that can be applied to disease modeling and therapeutic research using a variety of animals. In this review, we introduced and summarized animal models of human diseases and therapeutic studies based on the CRISPR-Cas9 system. Recently, a method of producing animal models using the CRISPR system was reported, which uses mainly microinjection, electroporation, and the newly developed GONAD method with fertilized 1-cell embryos. In particular, the electroporation of the protein or mRNA form of Cas9 enabled the easy and quick production of animal models. Moreover, the development of the GONAD method has simplified the animal modeling process without animal sacrifice.

Here, we also introduced studies of therapies that employed animal models of diseases using various delivery strategies, such as viruses, liposomes, peptides, or nanoparticles. However, the delivery strategies of the CRISPR system *in vivo* have not been fully validated regarding safety, accuracy, and efficiency. For clinical applications, further research will be needed to validate these delivery strategies. In addition, problems such as low HDR efficiency, off-target effects and immune rejection are steadily being raised. Several groups have recently pointed out that CRISPR-Cas9 causes unexpected large deletions and complex lesions in addition to predictable indels and conversion (Adikusuma et al. 2018; Kosicki et al. 2018). Thus, research aimed at overcoming these obstacles must be continued.
